# Sweet Success, Bitter Defeat: A Taste Phenotype Predicts Social Status in Selectively Bred Rats

**DOI:** 10.1371/journal.pone.0046606

**Published:** 2012-10-03

**Authors:** John M. Eaton, Nancy K. Dess, Clinton D. Chapman

**Affiliations:** 1 Department of Psychology, Occidental College, Los Angeles, California, United States of America; 2 Department of Psychology, Occidental College, Los Angeles, California, United States of America; Université Pierre et Marie Curie, France

## Abstract

For social omnivores such as rats and humans, taste is far more than a chemical sense activated by food. By virtue of evolutionary and epigenetic elaboration, taste is associated with negative affect, stress vulnerability, responses to psychoactive substances, pain, and social judgment. A crucial gap in this literature, which spans behavior genetics, affective and social neuroscience, and embodied cognition, concerns links between taste and social behavior in rats. Here we show that rats selectively bred for low saccharin intake are subordinate to high-saccharin-consuming rats when they compete in weight-matched dyads for food, a task used to model depression. Statistical and experimental controls suggest that differential resource utilization within dyads is not an artifact of individual-level processes such as apparatus habituation or ingestive motivation. Tail skin temperature measurements showed that LoS rats display larger hyperthermic responses to social interaction after status is established, evidence linking taste, social stress, autonomic reactivity, and depression-like symptoms. Based on regression using early- and late-competition predictors to predict dyadic disparity in final competition scores, we tentatively suggest that HiS rats emerge as dominant both because of an “early surge” on their part and because LoS acquiesce later. These findings should invigorate the comparative study of individual differences in social status and its relationship to mental and physical health.

## Introduction

The conventional view of taste as a chemosensory system tied exclusively to eating and drinking is yielding to evidence linking taste to a host of emotional, cognitive, and social processes. The new view emerging from laboratory animals and humans reflects more fully taste’s position, since early vertebrate evolution, at the “high stakes” interface between environmental toxins or nutrients and the internal milieu [1]. On this view, taste’s original evaluative function – to distinguish what is safe to swallow from what is potentially harmful and should be spit out – has been phylogenetically elaborated in multiple behavioral domains [2], [3]. Two lines of research support this view. The first concerns taste as a marker for individuals’ noningestive characteristics. Taste phenotype correlates in rats include startle amplitude, stress vulnerability, impulsivity, and responses to psychoactive drugs (see [4]). Examples in humans include negative affect, depression, prosociality, and alcohol abuse (e.g. [5], [6], [7]).

The second line of research concerns taste’s causal effects on noningestive processes. Sweet taste reduces pain and stress in rodents and humans and modulates future discounting in humans [8], [9], [10]. If such effects are explainable in terms of simple, phylogenetically old pathways, others are less so. For instance, administering a sweet taste softens moral judgment [11]. Conversely, bitter taste exacerbates moral disgust, more so among political conservatives [12].

That conservatism moderates a bitterness effect implies a functional pathway linking an evolutionarily old gustatory process to contemporary human sociopolitics. Such a pathway comports with conservatism’s basic biobehavioral underpinnings: It is heritable [13], correlates positively with disgust sensitivity [14], and is associated with temperament, curiosity, fear, and social dominance orientation (e.g. [15]). Rooting human sociopolitical theory in low organizational levels – in particular kinds of sensory systems, body morphometry and movement, experience-shaped epigenomes, and so on – typifies the emerging *psychology of embodiment* [16], [17]. This theoretical lens compels understanding of psychological processes not only through traditional exploration of “disembodied” processes for which the body is merely an input-output device, but also by directly engaging animals’ corporeal nature; an example is the study of mind through the lens of embodiment (*embodied cognition*). Meanwhile, other research is illuminating other species’ social complexity. Rodents and insects, for example, deploy prosocial behaviors contingently depending on their relationships to others (e.g. [18], [19]), and research on dispositional differences relevant to social life is increasingly comparative [20], [21]. The confluence of these literatures raises the question of whether taste is linked to social behavior in other species as it is in humans.

Here we show that a taste phenotype serves as a marker for social behavior in rats. The Occidental High- (HiS) and Low- (LoS) Saccharin Consuming lines have been selectively bred for more than 35 generations on the basis of voluntary saccharin intake (reviewed in [4]). The selection phenotype quickly distinguished the lines. The phenotype difference appears to reflect LoS rats’ weaker response to dilute sweet solutions and stronger response to adulteration of sweetness with bitterness, relative to HiS rats’ [22], [23]. Differential aversive responses to saccharin require a few minutes of taste experience [24], implicating experience-contingent, epigenetic processes in phenotype expression. Eventually, the lines diverged on acoustic startle amplitude, stressor impacts, impulsivity, and responses to ethanol, cocaine, and methylphenidate including self-administration, locomotor activation, withdrawal, and c-Fos expression in the nucleus accumbens [4], [25], [26]. Overall, relative to HiS rats, LoS rats are more anxious, more easily stressed, less impulsive, and less prone to psychoactive drug use. All previous research with these lines has examined them as individuals. The present study moved to the dyadic level, using resource competition under mild food deprivation [27], [28] to compare high- and low-saccharin rats’ proneness to social dominance or subordination. In addition, line differences on a noninvasive measure of autonomic reactivity was examined.

## Methods

### Ethics Statement

Care and use of the rats in the research reported here complied with Occidental College’s PHS Animal Welfare Assurance and was approved by the Occidental College Institutional Animal Care and Use Committee (IACUC).

### Rats

The rats were experimentally naïve adult males from 25 litters in Generations 37–39 of lines selectively outbred from Holtzman/Harlan Sprague-Dawley founding stock. The selection phenotype is assessed in a 24-hr two-bottle test (1 g/L saccharin solution versus tap water); the difference between saccharin intake and baseline water intake is expressed as a percentage of bodyweight (see [4] for details). The primary experiment involved 70 rats (35 weight-matched HiS/LoS dyads), and a follow-up control study involved 16 rats (HiS *n* = 8, LoS *n* = 8). Bodyweight did not differ significantly between lines in either study, *t*s <0.5, n.s.

### Apparatus and Procedure

#### Pre-competition measurements and manipulations

**Table 1** summarizes the primary experiment’s protocol. After brief handling sessions, an acoustic startle test was conducted in a commercial apparatus (San Diego Instruments, SR-Pilot Model). After a 3-min habituation period, 30 startle trials occurred on a 10-sec fixed-time schedule. Full-body startle reflex amplitude was recorded from a digital display (arbitrary units; see [29] for additional details). Reduction to 92% of free-feeding bodyweight through chow rationing began after startle testing. After preexposure to sweetened milk (100 g of sucrose per liter of whole milk) for 24 hr in homecages, rats were habituated to the competition apparatus by placing them in it alone for 5 min on two consecutive days. Each day, latency to first drink (sec) and total drinking time (sec) were recorded.

**Table 1:** Protocol for the primary study.

Day	Procedure
1–2	Briefly handle and record tail temperature
3	Briefly handle
4–5	Startle testing; begin food rationing for weight reduction
6…	Weight reduction, begin monitoring weight loss
7	Start sweetened milk consumption test
8	End sweetened milk consumption test
9	Monitor weight loss
10–11	Solitary habituation to apparatus and milk
12–21	Competitions 1–10

The number of days for weight reduction to criterion varied slightly 1–3 days) among squads, which were balanced for line.

Change in tail skin temperature (TST) indicates vasoconstriction and vasodilation and, putatively, is a peripheral marker of coping style [30]. TST was measured with a digital thermometer (±0.1°C, Omega Engineering HH63K, Stamford, CT, USA) before and after initial handling, startle testing, apparatus habituation, and early (third) and late (ninth) competitions. The thermocouple was embedded in an insulated wrap-around cuff that allowed the experimenter to quickly secure it to the tail base underside. The experimenter attached the cuff and placed the rat in a shoebox plastic cage near the homecage (initial measurement) or test apparatus until the reading stabilized (within a few seconds, maximum of 10 sec). This procedure occurred within one minute of the beginning and end of that day’s procedure.

#### Competitions

**Figure 1:**
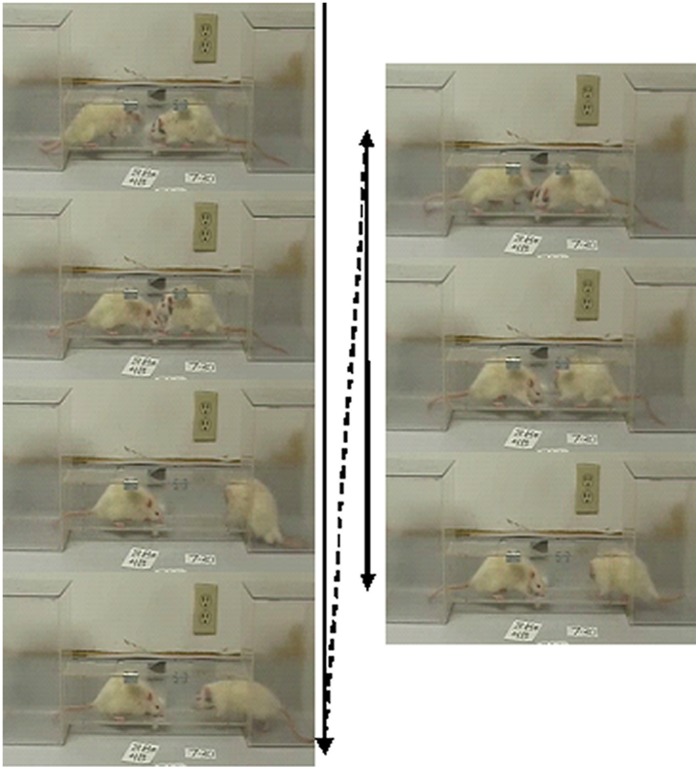
A sequence of frames from a late competition. The rat on the left (dominant) supplants the rat on the right (subordinate) at the milk cup. The latter retreats then returns; he is pushed back by the dominant rat and, unable to regain control of the milk cup, retreats again.

The clear acrylic competition apparatus with center reservoir for sweetened milk (**Figure 1**) and the competition procedure were based on work by Malatynska and colleagues [27], [28]. For each competition, a HiS rat and a weight-matched LoS rat were placed into different end boxes. They then were given simultaneous access to the alley by removal of barriers. Each dyad had one 5-min competition daily. In previous research, dominant-subordinate relationships, operationalized as difference between dyad members in total daily milk drinking time, began to stabilize after five competitions [27], [28]. Therefore, ten competitions were conducted with each of the 35 dyads. Competitions were videotaped and scored for each rat’s total daily drinking time. The primary observer was blind as to line until scoring was completed. A second blind observer scored a sample of 20 competitions, and interobserver reliability was high (Cronbach’s alpha = 0.98). Daily supplemental feeding occurred after all competitions were completed.

#### Control study: Solitary tests of drinking initiation

This follow-up study examined a potential individual-level line difference that could spuriously inflate the line difference in total daily drinking time: differential change in drinking initiation over multiple apparatus exposures. In the primary study, rats had two solitary apparatus exposures, and the line difference in total drinking time emerged after three competitions. Conceivably, LoS rats might take longer to start drinking than HiS rats after several apparatus exposures even if they are alone in the apparatus. If so, they would have less opportunity to sequester the milk cup for reasons unrelated to the presence of the HiS rat. To explore this possibility, LoS and HiS rats were tested individually in the apparatus for seven days, and drinking initiation was measured.

Experimentally naïve LoS and HiS rats (*n*s = 8) were preexposed to sweetened milk in homecages and then reduced to 92% of their free-feeding weight. On each of seven days, a rat was placed in the competition apparatus and remained there until he had drunk for a total of 5 sec. Drinking initiation latency was defined as the time it took for a rat to drink for 5 sec. Maximum session duration was 5 min. This measure improved on the latency measure used for solitary trials in the primary experiment because it required completion of a nontrivial amount of drinking. This time, a single lick or two did not count as drinking initiation.

#### Statistical analyses

Independent-groups statistics were used throughout to compare LoS and HiS rats. These tests are an obvious choice for solitary tests. Which tests are appropriate for dyadic competition phase data bears closer consideration. Repeated-measures tests usually are appropriate for matched pairs; usually, members of a pair are more similar to each other than to members of other pairs, and repeated-measures tests exploit that similarity to make the tests more sensitive to effects of interest. However, the nature of the intra-dyad relationship was at the heart of the present study. Dyad members were matched on bodyweight to make competition a “fair fight” – but dyadic competition can be expected ultimately to result in a *negative* intraclass correlation (ICC) (i.e. more drinking by one rat would be associated with less drinking by the other rat). If this were so, repeated-measures tests would be “unnecessarily conservative” [31]. *Changes* in ICC across competitions further violate assumptions of repeated-measures tests. Therefore, independent-groups tests were used, with nonindependence adjustments to *t*, *F*, and *df* (yielding *t’*, *F’*, and *df’*; [32]) in competition-phase analyses. Whereas *t’* and *F’* can be larger or smaller than unadjusted counterparts (depending on whether ICC is positive or negative), *df’* is always smaller than *df* and thus is a modestly conservative adjustment.

In addition, criteria from previous milk-competition research [27], [28] were used to evaluate whether each dyad’s dominant-subordinate relationship was “very strong.” A status difference was defined as very strong if three stringent criteria were met during the last five competitions: dyad members’ drinking scores on those five competitions differed significantly by a two-tailed *t*-test; the difference in average drinking time for the dyad members was at least 25% of the higher score; and no reversals occurred with respect to which dyad member drank more. The proportions of very strong relationships in which the HiS versus the LoS member was dominant were compared with a binomial test.

Supplemental analyses were performed to allow stronger inferences about the role of social interaction in drinking times. These analyses utilized Pearson’s *r* and Pearson-Filon *z* for pairwise correlations and a multiple regression to identify predictors of the line difference in drinking during the last competition.

All test statistics were evaluated for significance at α = .05, after adjustments for nonindependence [32] and α inflation (Greenhouse-Geisser corrections for trials effects, Bonferroni corrections for pairwise contrasts in ANOVAs).

## Results and Discussion

### Saccharin Phenotype Predicts Dominance

**Figure 2:**
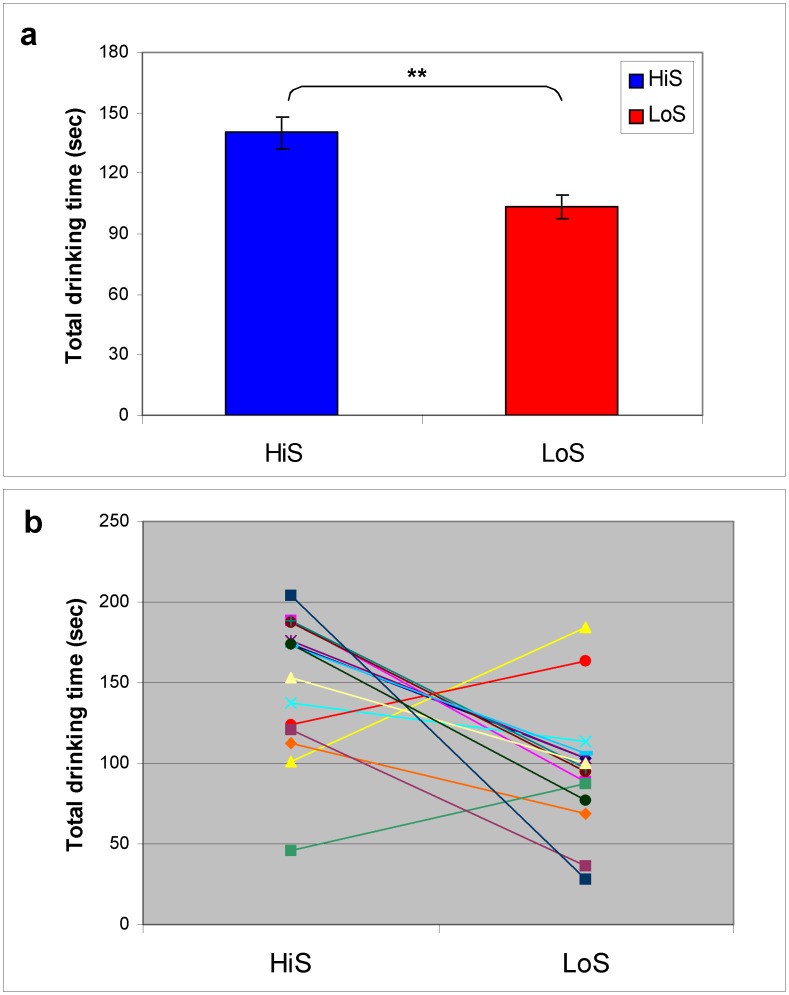
Total drinking time during the last competition by HiS and LoS rats. **a**, means (seconds ± s.e.m.) for all 35 dyads. Drinking time was higher in HiS rats than in LoS rats (*P*<.01, indicated by **). **b**, drinking times of individual rats in the 14 dyads (lines connect partners) meeting criteria for a “very strong” dominant-subordinate relationship (see text for criteria). The HiS rat was dominant in 11 of the 14 dyads.

With dominance defined as greater resource utilization during head-to-head competition, HiS rats ultimately dominated LoS rats. This conclusion is supported by several analyses. First, total drinking time during the last competition (Competition 10) was significantly greater in HiS rats than LoS counterparts (**Figure 2a**), *t’*(59) = 3.11, *P* = .003. Second, very strong relationships emerged in 14 of the 35 dyads (40%, which compares favorably to the 25–33% reported for randomly bred rats; [33]), and the HiS rat was dominant in 11 of those dyads (11∶3 split significant by binomial test, *P* = .029; **Figure 2b**). Applying less stringent criteria – whether a dyad meets the first or second criterion (19 dyads, with 15 HiS dominant), or simply which rat drank more in the last competition (25 of 35 were HiS) – also yields a significant line difference, *P*s ≤.005.

Some pushing and shoving, but not the biting typical in intruder paradigms (e.g. [34]), was observed (**Figure 1** **, Video S1**). The effect size for Competition 10 drinking time disparity was modest (mean line difference of ∼37 sec, η^2^ = 0.17). All rats sought and gained access to milk, and drinking times were not indirectly suppressed by extreme fear or submissive behaviors (e.g. freezing, boxing posture). Thus, social status took a form subtler than overt aggression, a form common within social groups – jockeying for access to a prized resource, with differences in assertion and acquiescence yielding differential access.

### Drinking was Sensitive to Social Context

Convergent evidence from a series of analyses indicates that the line difference in Competition 10 drinking time reflects, at least in part, social interaction. The lines drank comparable amounts of milk during homecage preexposure, *t*(68) = 0.30, n.s. The lines also had similar drinking latencies and total daily drinking times during solitary apparatus exposures (see **Table 2**; *t*s ≤1.4 for days separately or averaged, *F*s ≤2.4 for line effects in repeated-measures ANOVAs, all n.s.). That was true of HiS and LoS rats who later formed very strong status relationships as well as those who formed weaker relationships (all *t*s <1.6, n.s.). Furthermore, an ANCOVA on Competition 10 drinking time using solitary trial latency and drinking time as covariates still yields a significant line effect, *F’*(1, 53) = 7.98, *P* = .007.

**Table 2:** Individual-level characteristics of HiS and LoS rats.

Characteristic	HiS Mean ± s.e.m.	LoS Mean ± s.e.m.
Preexperimental bodyweight (g)	465±9	471±10
Acoustic startle amplitude (arbitrary units)*	216±22	322±29
Preexposure homecage milk intake (g)	128±4	127±4
Initial tail skin temperature (°C)	28.4±0.2	28.2±0.2
Solitary trials, daily latency to drink (sec)	110±17	114±16
Solitary trials, total daily drinking time (sec)	66±8	53±6
Saccharin phenotype score (Δ%)*	30±1	2±1

* Line difference significant, *P* ≤.05.

In addition, consistent with prior research [27], [28], dyad members had similar total daily drinking times during their early encounters. During the first three competitions, drinking time differed between lines only about as much as during solitary trials (∼13 sec), and none of those differences was significant. In contrast, HiS rats drank significantly more in six of the remaining seven competitions [mixed design ANOVA using 25 dyads, as data were unavailable for one competition for each of the other dyads; line×competition day interaction, *F’*(9, 381) = 3.04; *P*s ≤.02 for the significant Bonferroni-adjusted contrasts]. Given apparatus preexposure and relatively constant bodyweight, the emergence of a reliable line difference only after several competitions is more consistent with social influence than with changes in drinking motivation.

More evidence implicating social interaction in the line difference comes from examining correlations across the competition phase. Drinking times were reasonably stable within individuals; with one exception, all nine adjacent-day pairs (Competitions 1 and 2, Competitions 2 and 3, and so on) were significant for both HiS and LoS rats [*r*s >0.43, *P*s <.05]. The story is different at the dyad level. The LoS/HiS drinking time correlation was positive before status differences emerged [Competition 3; *r*(33) = 0.20], probably because dyad members were weight-matched. By the last competition (Competition 10), however, the drinking time correlation was *negative*, *r*(33) = −0.31: The more one dyad member drank, the less the other drank. This shift from a positive to an inverse relationship is significant [Pearson-Filon test for nonoverlapping dependent correlations, *z = *2.32, *P* = .02].

**Figure 3:**
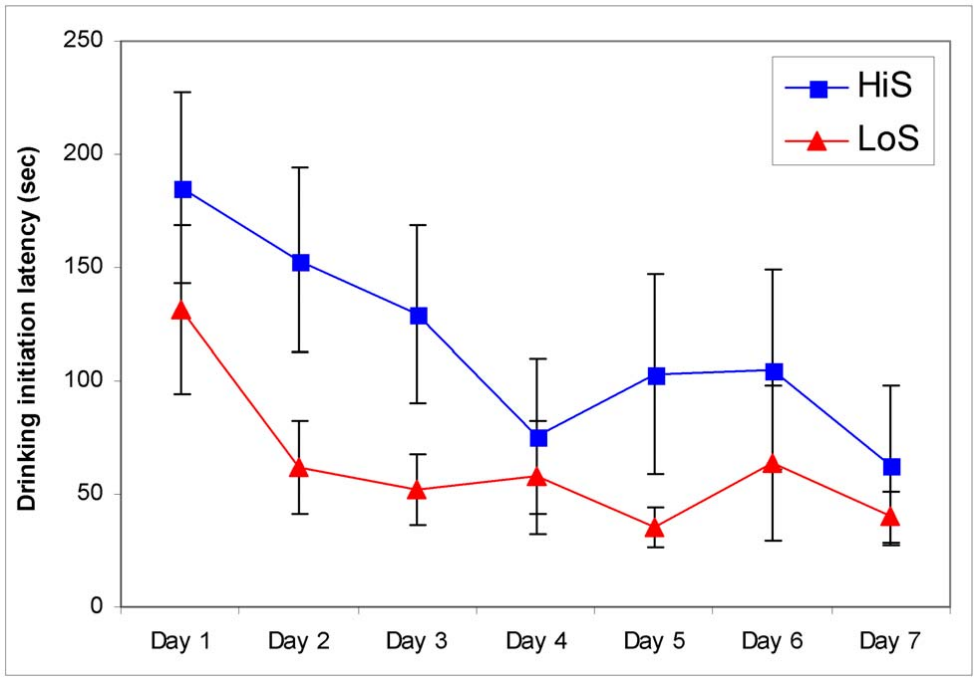
Control study data: Experimentally naïve, solitary rats’ latency to drink milk for a total of 5 s on each test day. Drinking initiation differed marginally across days and between lines.

The control study further undermines the idea that the lines differed in total drinking time because HiS rats are more motivated to drink milk rather than due to dyadic interaction. Among HiS and LoS rats tested individually for seven days (**Figure 3**), latency to initiate drinking trended downward across days [marginal day effect, *F*(5, 70) = 2.64, *P* = .08]. Importantly, HiS rats tended to take *longer* to drink for a total of 5 sec than did LoS rats [marginal line effect, *F*(1, 14) = 3.92, *P* = .07]. The direction of this nonsignificant line difference is not consistent with greater motivation to drink or early sequestration of the milk cup by HiS rats. This finding suggests that the liberal measure of drinking latency in the primary study was picking up “hit and run,” unsustained milk cup visits by some HiS rats.

If the lines’ similarity in motivation to drink sweetened whole milk seems surprising given selection on a sweetener-intake phenotype, it is no real puzzle: While freely feeding LoS rats do consume less “free” dilute sweet solution and earn fewer sucrose pellets in an operant task [22], [35], they are more sensitive to threats to metabolic homeostasis than are HiS rats [36]. For example, at a reduced bodyweight, LoS rats work harder to defend a constant eating rate than do HiS rats [37]. Similarly, LoS rats self-administer less cocaine on average but behaviorally regulate dose more precisely across reinforcement contingencies than do HiS rats [38]. The present data from reduced-bodyweight rats indicate that in this paradigm, any enhancement of sweet taste incentive among HiS rats is offset by LoS rats’ greater motivation to avoid negative energy balance.

The blend of individual and dyadic level processes through which HiS rats gain priority access to milk remains to be determined. Future research can evaluate specific models of dyadic interaction, such as latent dyadic, actor-partner interdependence, and slopes-as-outcomes or growth curve [39] models. In the meantime, a final post-hoc analysis of these competition data yields a tentative, atheoretical suggestion: HiS rats may emerge as dominant because of an “early surge” on their part to which LoS rats ultimately acquiesce. Competition data were subjected to multiple regression to evaluate the variance in dyads' ultimate drinking disparity score (Competition 10 total drinking time for HiS member – LoS member) accounted for by four predictors: early (Competition 3) and late (Competition 9) drinking times for HiS and for LoS dyad members. These competitions were, respectively, before and after a significant line difference in total daily drinking time emerged. Two predictors contributed uniquely to variance in Competition 10 disparity score: HiS rats’ drinking time in the early competition [standardized partial β = 0.49, *t*(30) = 3.65, *P = *.001] and LoS rats’ drinking time in the late competition [standardized partial β = -0.31, *t*(30) = 2.02, *P = *.05]. Thus, HiS and LoS dyad members may both contribute to the establishment of their status relationship, but their roles may vary over time: Early assertion by HiS rats (positive predictor of last disparity) may contribute to their LoS social partners eventually deferring to them (negative predictor of last disparity). Whether this is the case requires additional research and statistical designs that allow stronger causal inferences about dyadic dynamics.

### Selection Phenotype and Correlates

**Figure 4:**
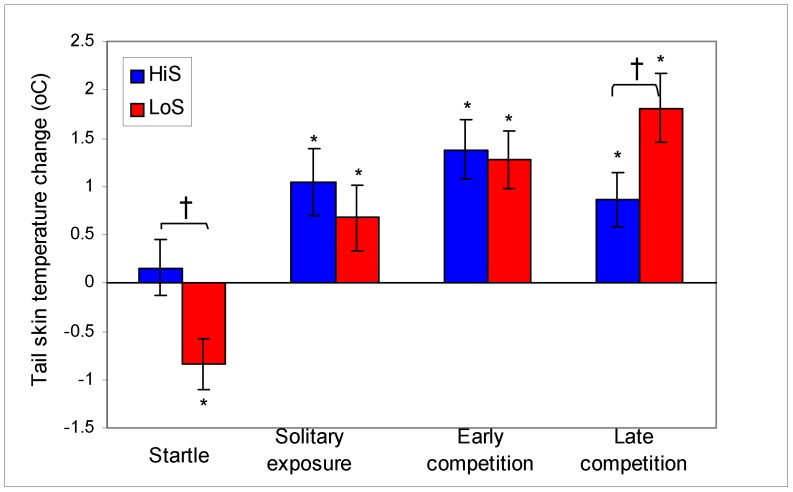
Tail skin temperature (TST) changes (mean °C ± s.e.m.) during four challenges. Significant changes (vs 0) are marked with an asterisk (*). Significant line differences are marked with a dagger (†).TST dropped during startle testing only in LoS rats. Hyperthermic responses to the competition apparatus (Solitary exposure) and to Competition 3 (Early competition) did not differ between lines but were larger in LoS rats during Competition 9 (Late competition). Note: TST response to startle was measured in 23 dyads; they did not differ from the other dyads in the other tests with respect to line effects.

Tail skin temperature (TST) changes from before to after various challenges are shown in **Figure 4**. These data were examined for line differences (independent *t* tests) and for absolute changes from 0 (one-sample *t* tests). The hypothermic response to the startle test differed between lines, *t*(44) = 2.52, *P* = .02; the drop in TST was significant only among LoS rats, *t*(22) = 3.13, *P = *.005. The novel apparatus and an early competition (Competition 3) produced hyperthermia in both lines [first solitary trial, *t*(34) = 3.00 and 2.01 for HiS and LoS, respectively, *P*s ≤.05; early competition, *t*(34) = 4.45 and 4.32 for HiS and LoS respectively, *P*s <.001], and did so to a comparable extent [line difference, respectively, *t*(68) = 0.74 and *t’*(68) = 0.23,^1^ n.s.]. Both lines also displayed a hyperthermic response to a late competition (Competition 9) [*t*(34) = 3.06 and 5.07 for HiS and LoS, respectively, *P*s <.01]; however, the response was significantly larger among LoS rats, *t’*(66) = 2.17, *P* = .03. Thus, line differences in TST responses are stressor specific [30]: LoS rats show an exaggerated hypothermic response to startle testing and an exaggerated hyperthermic response to competitions after status relationships are established. LoS rats’ exaggerated response to late dyadic interaction might generalize to other stressors [40].

Assessment of acoustic startle, an established phenotypic correlate (e.g. [29]), yielded the usual hyperstartle in LoS rats, *t*(68) = 2.89, *P* = .005. A two-bottle water vs. saccharin test conducted after the conclusion of the study confirmed the selection phenotype, *t*(68) = 16.83, *P*<.001. (See **Table 2**).

### Conclusion

This study provides convergent evidence that a taste phenotype is a marker for behavioral and physiological responses to social interaction. With dominance and subordination defined in terms of differential utilization of a prized food resource, a predisposition to lower voluntary saccharin intake predicted lower social status. Our rats did not engage in serious fighting, but encounters with a conspecific clearly can pose a threat. One way of interpreting the regression results – that HiS rats get the jump on LoS rats, and LoS rats ultimately acquiesce – may speak to different coping styles. Korte et al. [41] distinguished a “Hawk” style, characterized by bold, rapid, risk-prone, fight-flight responses, from a cautious, slow, risk-averse, lay-low style (“Dove”). Applying their scheme to our rats’ response to a conspecific rival, HiS rats may be Hawks, LoS rats Doves. Consistent with the implication that Dove-ish anxiety mediates deference as a coping strategy, anxiolytic drugs increase sweetened milk intake by subordinate rats in competitions [42].

Oversimplifying LoS rats’ social fate, however, would be a mistake. Competition for palatable food is motivationally complex, arguably more complex than resident-intruder or other social defeat paradigms. Elsewhere [37], we proposed that some behavioral differences between LoS and HiS rats can be conceptualized using a cognitive-forager model [43]. This model captures the approach-avoidance conflict faced by opportunistic omnivores such as rats and humans – the need to seek and exploit food resources while contending with competitors and predators. In this framework, LoS rats’ deference here might have been conditional on, for instance, mild deprivation, reliable supplemental feedings, and a weight-matched rival; under these parameters, the benefits of yielding the milk cup to some extent might outweigh the costs. We ought not assume that LoS rats, even if they do tend toward a Dove-ish or reactive coping style, would defer to HiS rats under all circumstances [44], [45].

Extending this logic, it would be wrongheaded to think of LoS rats’ acquiescence (or HiS rats’ assertion) as “adaptive” or “maladaptive” based on these findings. The fitness consequences of deference and assertion, in the past and the present, would depend on circumstances. How serious is negative energy balance? How limited are food resources (and mates), for how long? How dangerous is the rival? How heavy is predator pressure? Fitness consequences also would depend on how strategically a rat responded to changing circumstances. Additional research using multiple dominance paradigms and parametric variations (e.g. [46]) would provide the opportunity to see whether LoS and HiS rats respond in a rigid or flexible way to social stress.

That said, the stress of *chronic* social subordination clearly compromises physical and mental health in humans and other species ranging from shrews to salmon [47], [48], [49]. Thus, individual differences in proneness to emerging from social interactions as dominant or subordinate deserve more attention in rodent models, especially models of the “common cold” of psychopathology – depression [49], [50]. One avenue of study would examine taste phenotypes and the epigenetic and neural mechanisms through which they are expressed, to identify common pathways related to health outcomes. Another, in line with work by Malatynska and colleagues [28], [34] would concern antidepressant drug efficacy, particularly the efficacy of candidates for rapid antidepressant activity [51] in alleviating low social status. Working with rodent lines prone to forming strong dominant-subordinate relationships, such as the HiS/LoS lines, would facilitate such research by increasing the proportion of dyads displaying strong relationships and by modeling double-hit (vulnerability×environmental adversity] pathogenesis [52], [53].

The present findings add to a contemporary literature that calls for reexamination of prior research on individual differences in taste as well as formulation of new studies of the genetic, epigenetic, and functional relationships between taste and noningestive phenomena. That the functioning of a relatively primitive chemosensory system has been linked to social relationships in humans [6], [11], [12] and, now, in rats signals that the time has come for the nascent psychology of embodiment to encompass diverse species [17]. Basic sensorimotor processes may be linked to higher order affective and cognitive processes in rats and humans via pathways conserved in mammalian evolution, or via quite different mechanisms that evolved in relation to group living and/or omnivory (convergent evolution, facultative adaptations), or a mix of these. These accounts can be best examined through collaboration among researchers with expertise in behavioral neuroscience, epigenetics, and comparative, cognitive, and social psychology.

## Supporting Information

Video S1 **This edited video clip illustrates typical encounters at the milk cup between a dominant rat (on the left) and a subordinate rat (on the right).** Frames from this clip are shown in **Figure 1**. Encounters of this kind over the 5 min competitions generate a significant difference between HiS and LoS rats in total drinking time.
(WMV)
