# Correction: Sweet Success, Bitter Defeat: A Taste Phenotype Predicts Social Status in Selectively Bred Rats

**DOI:** 10.1371/annotation/980a13fe-34d4-4d8a-89ee-65fa88703fcd

**Published:** 2013-08-13

**Authors:** John M. Eaton, Nancy K. Dess, Clinton D. Chapman

Additional funding from Occidental College was incorrectly omitted from the Funding Statement. This funding was given through the VanderWeele Student Research Fund. 

